# Greater rate of cephalic screw mobilisation following proximal femoral nailing in hip fractures with a tip–apex distance (TAD) and a calcar referenced TAD greater than 25 mm

**DOI:** 10.1186/s13018-018-0814-1

**Published:** 2018-05-02

**Authors:** Rocco Aicale, Nicola Maffulli

**Affiliations:** 1 Department of Musculoskeletal Disorders, Faculty of Medicine and Surgery, University of Salerno, Salerno, Italy; 2 Centre for Sports and Exercise Medicine, Barts and The London School of Medicine and Dentistry, Mile End Hospital, 275 Bancroft Road, London, E1 4DG England

**Keywords:** Hip, Fracture, TAD, CalTAD, TADcalTAD

## Abstract

**Background:**

To ascertain whether the tip–apex distance (TAD), calcar referenced TAD (CalTAD), and the sum of both (TADcalTAD) are predictive measurements of mobilisation of the cephalic screw in patients with trochanteric hip fractures.

**Methods:**

Between 2014 and 2015, 68 patients (mean age 86 years, 45 females, 23 males) with a trochanteric hip fracture underwent intramedullary nailing. The TAD and CalTAD were measured, and for each parameter, we calculated sensitivity, specificity, positive predictive value (PPV) and negative predictive value (NPV).

**Results:**

There is evidence of a statistically significant association between a TAD and CalTAD greater than 25 mm and a TADcalTAD greater than 50 mm and mobilisation of the cephalic screw. All measurements have similar sensitivity, but the TAD presents the highest specificity (*p* < 0.01).

**Conclusion:**

To avoid the risk of mobilisation of the cephalic screw and possible subsequent failure of the construct, surgeons should strive for a TAD and CalTAD less than 25 mm and a TADcalTAD less than 50 mm when using intramedullary fixation.

## Background

Intertrochanteric hip fractures continue to pose problems for orthopaedic surgeons, as their incidence continues to rise to epidemic proportions [1, 2]. Over 700,000 deaths are estimated to occur annually worldwide following hip fractures [2], the highest surgical mortality of any orthopaedic operation [3, 4].

According to the World Health Organisation, hip fractures are associated with 20% 1-year mortality and 50% loss of function [5]. Italy has the lowest 1-year mortality rate (mean 19.1%) and the highest length of hospital stay (23.3 days) when compared with other European countries [6].

Sliding hip screws (SHS) and intramedullary (IM) devices are commonly used in the surgical management of unstable intertrochanteric (IT) and subtrochanteric (ST) fractures [7]. Outcomes are similar for both devices in intertrochanteric patterns [8], but failures continue to occur despite improvements in the devices and surgical techniques.

Baumgaertner et al. described the measurement of the “tip to apex distance” (TAD) in 1995 as a means of assessing the placement of a dynamic hip screw within the femoral head [9]. The TAD is calculated by adding the distance from the tip of the hip screw to that of the apex of the femoral head on the anteroposterior (AP) and lateral views. The target maximum distance was set at 25 mm, as the authors reported no failure of fixation from mobilisation of the sliding hip screw in the femoral head in the patients with a TAD lower than this distance [10].

The original description of measurement of the TAD was by direct measurement from printed hard copy radiographs [9]. The use of digital picture archives and communication systems is accurate and reproducible to measure the TAD for research and audit purposes [11].

An alternative to the centre-centre position is the low-centre position, in which the tip of the cephalic screw is placed in the lower 1/3 of the femoral head on the AP intraoperative fluoroscopic view and in the centre of the femoral head on the lateral view. Placement of the lag screw in this position may well lead to a tip–apex distance measurement that is greater than the 25 mm suggested by Baumgaertner et al. [9]. A cadaveric biomechanical study showed equal if not superior stability of the low-centre position with a tip–apex distance greater than 25 mm as the centre-centre construct with the currently accepted optimal tip–apex distance less than 25 mm, but this result is influenced by the quality of the bone through which the lag screw is placed [12].

The most common mode to mobilisation of the cephalic screw through the femoral head occurs when the fracture collapses into varus [13, 14]. There is an increased risk of cephalic screw mobilisation in older or osteoporotic patients, those with unstable fractures, and after poor reduction [15, 16]. Other factors which lead to cephalic screw mobilisation include implant angle and the position of the lag screw in the femoral head [14].

More recent studies have been conducted in dynamic hip screw (DHS) or have included DHS and intramedullary (IM) devices [16–19], but there is a paucity of literature on proximal femoral nailing cutout or mobilisation. In this context, the term “mobilisation” refers to abnormal postoperative motion of the cephalic screw or the nail with an increase of TAD greater than 3 mm. To the best of our knowledge, only Geller et al. [20] and Lobo-Escolar et al. [21] have reported that a TAD greater than 25 mm is a predictor of cephalic screw mobilisation in elderly patients with hip fractures treated with proximal femoral nailing.

Kashigar et al. [22] have reported an association between CalTAD and cephalic screw mobilisation of cephalic screw in elderly patients with hip fractures treated with femoral nailing. The CalTAD can be calculated using the same measurement technique of the TAD in the lateral view but differs in the anteroposterior (AP) view. Indeed, while for the TAD the measurement in the AP view is performed by identifying the apex of the femoral head using a guideline through the midline of the femoral head (in mm), for the CalTAD in the AP view, the measurement (in mm) is performed by moving this line to be tangent to the medial cortex of the femoral neck. The TAD in the lateral view is added to both these measurements to obtain TAD and CalTAD, respectively.

In this study, we introduce another parameter given by the sum of TAD and CalTAD, which we named TADcalTAD.

We used the tip–apex distance, the calcar referenced TAD and the TADcalTAD following intramedullary nail fixation of extracapsular hip fractures, to ascertain whether these measurements were associated with the risk of mobilisation of the cephalic screw.

## Methods

Fracture patterns were classified according to the systems of Muller et al. [23] and Evans [24] as modified by Kyle, Gustilo and Premer [25]. Based on the Orthopaedic Trauma Association (AO) fracture classification, the intertrochanteric fractures were classified as 31.A1 (*N* = 18; (26.5%)), 31.A2 (*N* = 43, 63.2%) and 31.A3 (*N* = 7, 10.3%) [26].

We included all patients with a traumatic trochanteric hip fractures treated by Zimmer Natural Nail System (CephaloMedullary Femoral Nail; Zimmer; Warsaw; IN, USA) or TrigerIntertan Nail (Smith&Nephew, Memphis, TN, USA), who had anteroposterior and lateral plain radiographs, complete clinical records and a minimum follow-up of 3 months. No patients were excluded based on age or other medical comorbidity. The selected patients were operated between 2014 and 2015 by different surgeons, and of 173 consecutive patients treated with proximal femoral nailing, 68 met these criteria. The choice of nail to use was left to the operating surgeon. All patients followed the same postoperative rehabilitation protocol until hospital discharge and were discharged to the same rehabilitation institution after an average of 14 postoperative days. All reductions of hip fracture were performed before the operation started on a dedicated fracture table and evaluated using fluoroscopy. The quality was classified in good, acceptable and poor according to the available scientific evidence [9].

The position of the lag screw in the femoral head was recorded by dividing the femoral head into nine zones resulting from the combined permutations of the lag screw in anteroposterior and lateral views. To define the boundaries of the nine zones, the femoral head was divided into thirds on both the AP and lateral views.

**Fig. 1:**
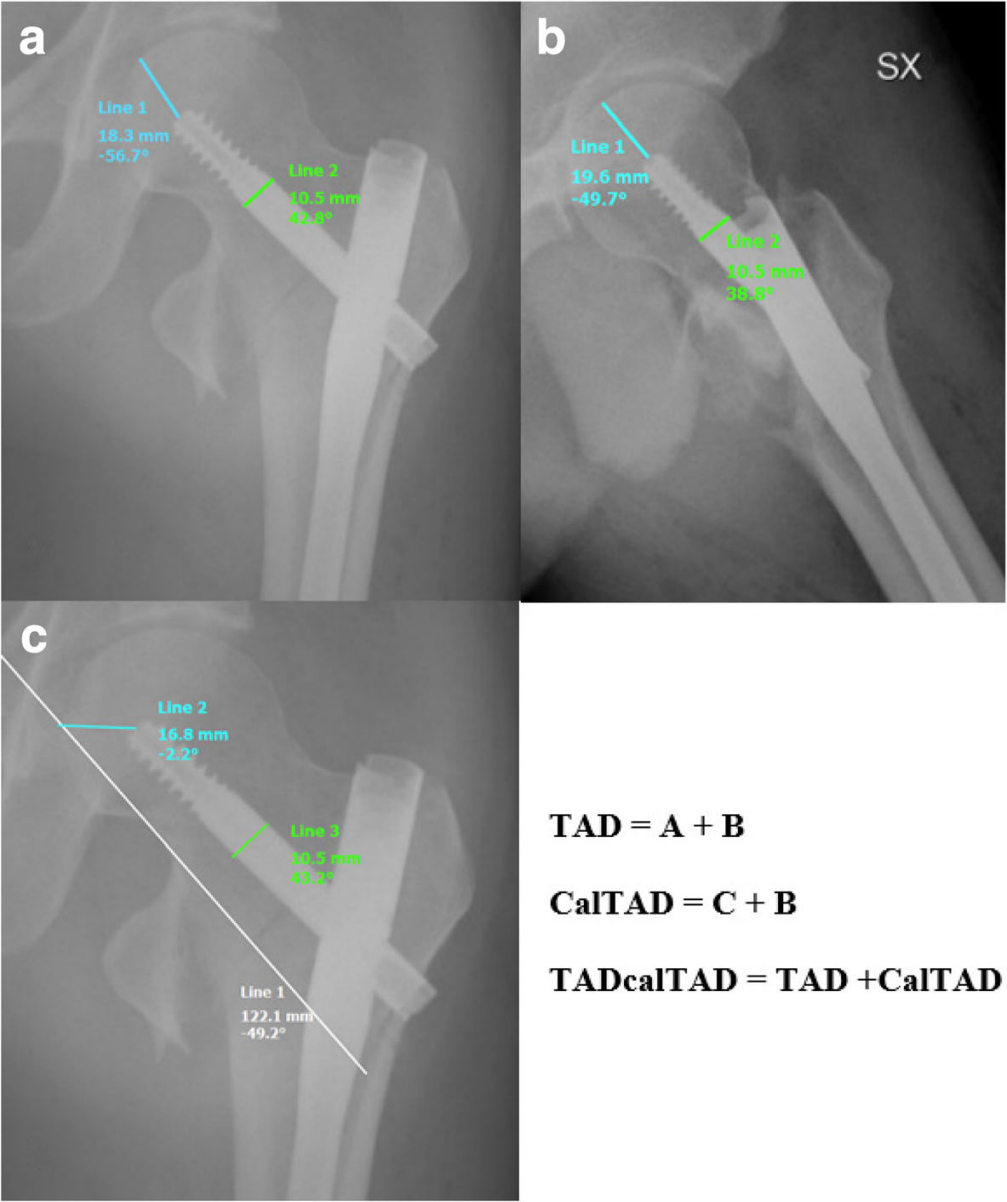
Schematic drawing of TAD measurement (**a, b**), CalTAD measurement (**c**) and TADcalTAD

The calculation of TAD has previously been reported in detail [9]. It is the sum of the distances from the tip of the lag screw to the apex of the femoral head on AP and lateral radiographs. We adjusted for radiographic magnification using the known diameter of the hip screw. The CalTAD is the sum of a TAD in the lateral view and the distance, in the AP view, between a line tangent to the medial cortex of the femoral neck and the tip of the lag screw. The TADcalTAD is the sum of the TAD and the CalTAD (Fig. 1).

Data collected included patients’ age at surgery, gender, fracture type, operative side, surgeon, type of implant, TAD and a minimum 3-month postoperative ambulatory status. Fracture type, TAD and CalTAD were determined using preoperative and postoperative anterior-posterior (AP) and lateral hip digital radiographs.

All records containing clinical and radiographic information of patients, admitted with a hip fracture to the San Giovanni di Dio e Ruggi d’Aragona Hospital of Salerno (Italy) during the years 2014 and 2015, were retrieved from the hospital database.

All images were exported into the Surgimap Software (Nemaris Inc., New York, NY, USA) to measure the TAD and CalTAD, for each radiograph, knowing the diameter of the cephalic screw (10.05 mm). Each measurement per each set of radiographs was repeated in a blinded fashion after 1 month in the same way comparing the two sets of measurements using Cohen’s kappa test to calculate the intra-tester reliability. Using the mean values of TAD and CalTAD, we then calculated the TADcalTAD.

Sensitivity, specificity, positive predictive value (PPV) and negative predictive value (NPV) were calculated for each parameter, and the specificity was compared between each measurement using Fisher’s exact test.

All data were entered in the Microsoft Excel software. We used the tool for Fisher’s exact test to analyse the association between TAD, CalTAD and TADcalTAD and cephalic screw mobilisation of the cephalic screws.

We used Fisher’s exact test because the contingency tables had a small sample size. The usual rule for deciding whether the chi-squared approximation is good enough is that the chi-squared test is not suitable when the expected values in any cells of a contingency table are below 5. In this way, the calculations are exact, rather than relying on an approximation that becomes exact in the limit as the sample size grows to infinity.

## Results

A total of 68 eligible patients received intramedullary nail fixation during the study period. Seventy-eight precent (*N* = 53) were treated with Zimmer Nails, and 22% (*N* = 15) were treated with Intertan nails.

Cohen’s kappa test was used to calculate the intra-tester reliability on the 1-month TAD and CalTAD repeated measured per each radiograph measurement. There was good intra-tester reliability (0.82 and 0.84, respectively). For the purposes of this article, we used the average between the first and the second measurement and the sum of both.

Fractures were classified according to the Orthopaedic Trauma Association (AO) fracture classification [26] into 18 A1 (26.5%), 43 A2 (63.2%) and 7 A3 (10.3%).

According to available scientific literature, the quality of the reductions was classified in good, acceptable and poor, resulting in a good (79%, *N* = 54) or acceptable (21%, *N* = 14) reduction for all patients enrolled in this study.

**Fig. 2:**
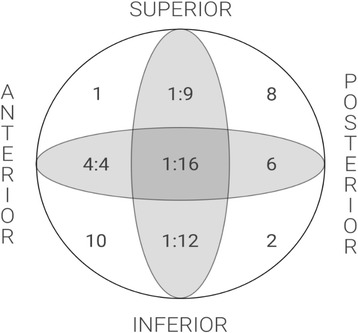
The distribution of lag screw positions in the femoral head and numbers of mobilisations for each position

The majority of the lag screws was implanted in the centre-centre, centre-inferior or inferior-posterior positions (16, 12 and 10, respectively); in the four patients in whom mobilisation occurred, the cephalic screw had been implanted in the centre-anterior position (Fig. 2). The small number of patients does not allow meaningful analysis within each of the nine zones of the femoral head.

Of 68 patients, 34% (*N* = 23) were males and 66% (*N* = 45) were females, with a mean age of 86 ± 19 years. The mean TAD and CalTAD for the whole population was 26.73 ± 7.97 mm and 26.37 ± 4.96 mm, respectively. The mean TAD of those patients who did not experience mobilisation of the cephalic screw was 25.98 ± 7.97 mm; the CalTAD for the same group was 25.83 ± 5.23 mm. The mean TAD of those patients who experienced mobilisation of the cephalic screw was 34.11 ± 6.67 mm, while the CalTAD for the same group was 31.04 ± 3.59 mm.

**Fig. 3:**
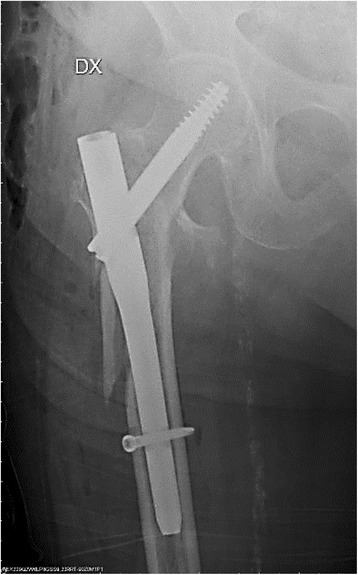
Radiograph of Zimmer nail with cephalic screw with TAD lower than 25 mm for 31-A3

**Fig. 4:**
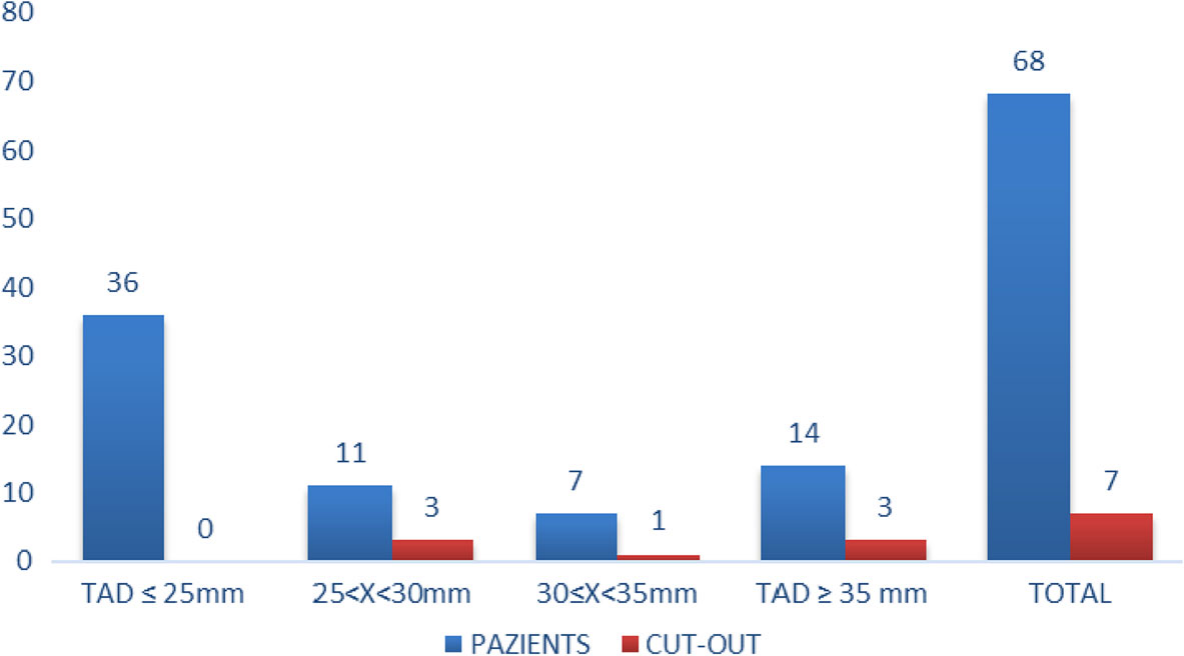
Mobilisation of the cephalic screw according to tip–apex distance (TAD) (mm)

A total of 53% (*N* = 36) of the patients had a TAD < 25 mm, and none of these experienced mobilisation of the cephalic screw (Fig. 3). On the other hand, 47% (*N* = 32) of the patients had a TAD > 25 mm and 21.8% (*N* = 7) of these showed cephalic screw mobilisation of the cephalic screw (Figs. 4, 7 and 8).

Of the entire population studied, 22% (*N* = 15) was treated with Intertan nails. Of these, 6 patients (8.82% of the entire population) had a TAD greater than 25 mm and 1 patient demonstrated mobilisation of the cephalic screw (Fig. 8).

Of the entire population studied, 78% (*N* = 53) was treated with a Zimmer Natural nail. Of these, 26 patients (38.23% of the entire population) had a TAD greater than 25 mm and 6 of the 7 patients who had mobilisation of the cephalic screw are in this subgroup.

**Fig. 5:**
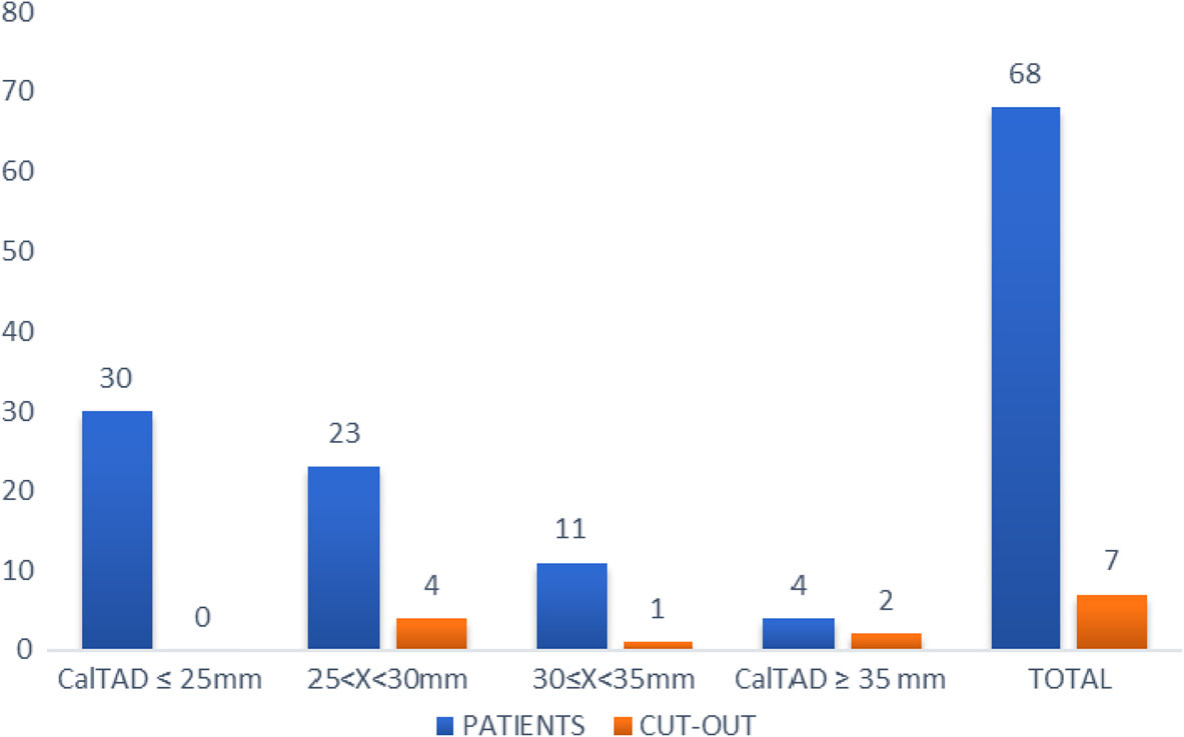
Mobilisation of the cephalic screw according to calcar referenced tip–apex distance (CalTAD) (mm)

The available literature does not offer a cutoff value for the CalTAD. In the present study, we considered 25 mm (as for TAD) the limit for predictive measurement of mobilisation. Fifty-six percent of the entire population (*N* = 38) presented a CalTAD greater than 25 mm, and all the patients with mobilisation of the cephalic screw are in this group (Fig. 5).

Only in two patients was the TAD greater than 25 mm but the CalTAD was not: in none of these did mobilisation occur. There were 8 patients with a TAD less than 25 mm and a CalTAD greater than 25 mm: in none of these did mobilisation occur. For the rest of the population, both TAD and CalTAD were greater 25 mm.

**Fig. 6:**
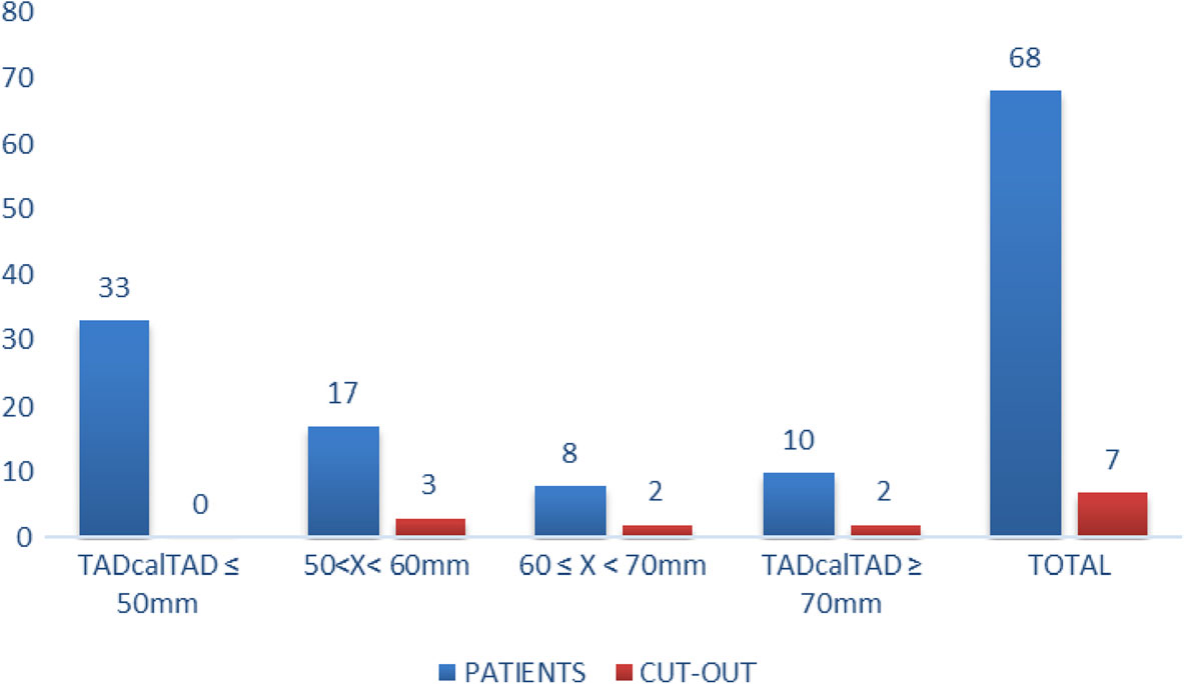
Mobilisation of the cephalic screw according to TADcalTAD (mm)

We considered a TADcalTAD of 50 mm as the limit to be predictive of mobilisation. Of the entire population studied, 51% (*N* = 35) had a TADcalTAD greater than 50 mm, and all patients who experienced mobilisation of the cephalic screw were in this group (Fig. 6). In 1 patient, the TAD was greater than 25 mm and the TADcalTAD was less than 50 mm, while in 4 patients, the CalTAD was greater than 25 mm and the TADCalTAD was less than 50 mm. In 4 patients, the TADcalTAD was greater than 50 mm with a TAD less than 25 mm and a CalTAD greater than 25 mm, while in 1 patient, the TADcalTAD was greater than 50 mm with a CalTAD less than 25 mm and a TAD greater than 25 mm.

These three parameters have the same sensibility: all the patients who experienced cephalic screw mobilisation were, in each group, over the cutoff limit per each parameter. The TAD presents a statistically significant higher specificity than the CalTAD and TADcalTAD (59.1 vs 49.2%, *p* < 0.001; 59.1 vs 54.1%, p < 0.001). The TADcalTAD presents a statistically significant higher specificity than CalTAD (54.1 vs 49.2%, p < 0.001). The 95% confidence intervals (CI) per specificity and sensibility, PPV and NPV are reported in Table 1.

**Table 1:** Measurements comparison between TAD, CalTAD and TADcalTAD

	Specificity		Sensitivity		PPV	VPN
	Estimate	95% CI	Estimate	95% CI		
TAD	0.59	0.42–0.76	1	–	0.22	1
CalTAD	0.492	0.32–0.66	1	–	0.18	1
TADcalTAD	0.541	0.37–0.71	1	–	0.2	1

*CI* confidence interval, *PPV* positive predictive value, *VPN* negative predictive value

Fisher’s exact test showed evidence of a statistically significant association between a TAD and CalTAD greater than 25 mm and mobilisation of the cephalic screw (*p* = 0.0035). The same test showed evidence of a statistically significant association between a TADcalTAD greater than 50 mm and mobilisation (*p* = 0.0035).

There was no statistically significant association (*p* < 0.05) between mobilisation and age, sex, side of fracture, implant type or operating surgeon.

A post hoc power analysis performed to evaluate the statistical power of the tests we have used showed that the present investigation had a 0.84 power to detect differences between the group with TAD greater than 25 mm and that with a TAD lower than 25 mm. This confirms that the sample enrolled in the present study is adequate for the purposes of our investigation.

## Discussion

In the USA, more than 90% of all proximal femoral fractures occur in patients over the age of 50. The occurrence of hip fractures doubles for each decade over 50 years [27]. With the increase of ageing population and a limited amount of healthcare resources, it will be increasingly important to avoid complications when operating hip fracture patients. These patients are exceedingly fragile, as evidenced by the occurrence of the fracture itself. Most of these patients will be unable to endure a second operation, or tolerate prolonged physical therapy. They will probably be discharged to a rehabilitation institution for an extended period of time [27–30]. Even after one operation, there is a 12-month mortality of 35% for men and 22% for women [31, 32].

The treatment options available for hip fractures include plates, nails and screws [33]. Fixation with a fixed-angle device, such as a sliding hip screw (SHS) plate or a cephalomedullary (CM) nail, is the preferred treatment option for intertrochanteric fractures of the hip [34]. CM nailing has proved especially popular for the treatment of unstable intertrochanteric and subtrochanteric hip fractures [35]. Indeed, patients experience an improved outcome when an intramedullary nail is used to treat their unstable intertrochanteric fracture [36]. CM nailing, compared with SHS, is associated with a shorter operating time, reduced intra-operative blood loss, and improved walking ability in unstable hip fractures [37, 38].

The TAD is the sum of the distance from the tip of the screw to the apex of the femoral head on anteroposterior and lateral views. Only two previous studies found high TAD values to be a significant predictor of mobilisation of IM nails [20, 21]. Our study further confirms this finding.

The tip–apex distance should guide the surgeon at the time of surgical fixation of the fracture. This would usually require estimation of the distance by sight, from the image intensifier. The accurate identification of the tip–apex distance by eye in cephalic screw placement has been demonstrated as greater than 80% in consultants and registrars who are aware of the concept [10].

The major limitations of the present study are its retrospective nature and the relative small number of patients included. Our study, nevertheless, includes a satisfactory number of patients with adequate follow-up, considering that, usually, the large majority of these patients fails to return for follow-up because of a multitude of factors when they are transferred to intermediate base facilities after hospital discharge. Additionally, the mortality rate is likely to be high. This study is one of the few that evaluates the association between TAD and CalTAD, with values greater than 25 mm, and mobilisation of the cephalic screw in patients treated only with intramedullary nailing, confirming this association and the fact that, with a greater distance, there is a greater chance of cephalic screw mobilisation (Figs. 4 and 5).

**Fig. 7:**
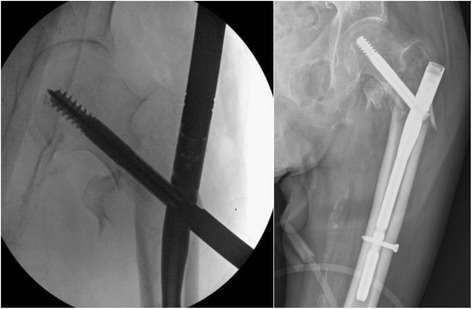
Radiographs of Zimmer nail with cephalic screw with TAD between 25 and 30 mm during surgery and after 5 months with mobilisation of the cephalic screw for a fracture 31-A2

**Fig. 8:**
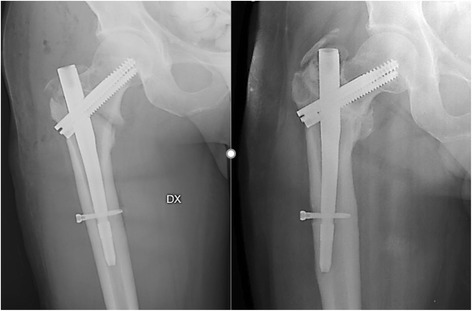
Radiographs of InterTan nail with cephalic screw with TAD greater than 35 mm during surgery and after 5 months with valgus mobilisation of the intramedullary nail for a fracture 31-A1

The high incidence of mobilisations in intertrochanteric fractures with TAD and CalTAD greater than 25 mm in this study is remarkable. It stresses the importance of accurate surgical technique to prevent failure of fixation (Figs. 7 and 8). The chance of mobilisation is probably exacerbated by the poor bone quality in the region surrounding the lag screw postoperatively, and patients who have significantly multi-fragmented intertrochanteric fractures may be more prone to mobilisation [39]. We are conscious of the fact that we did not evaluate the bone mineral density (BMD) in our patients, but we point out that there is no agreement regarding values of BMD that may be used to indicate a higher risk of mobilisation in osteoporotic bone [40] Furthermore, the TAD has been shown to be an independent predictor risk factor of cephalic screw mobilisation after internal fixation [9, 41], while the Singh Osteoporosis Index does not exhibit any significant relationship with the rate of cutout [22]. If revision surgery becomes necessary, a high mortality, or at the very least a high rate of in-hospital morbidity, and increasing costs are likely [20].

The magnitude of instability is different in types A1, A2 and A3 of the AO classification, but there are no studies evidencing different rates of mobilisation or cutout in elderly patients treated with intramedullary nail for hip fracture based on the association between AO fracture classification and TAD or CalTAD.

This study used a minimum value of CalTAD and considered the sum of TAD and CalTAD as a possible predictive measurement of mobilisation. The TAD, CalTAD and TADcalTAD are all able to predict the mobilisation of the cephalic screw. The TAD is more specific than the other two measures; the TADcalTAD is more specific than the CalTAD. In addition, the TAD has a higher PPV than CalTAD and TADcalTAD, but the latter presents a PPV higher than the CalTAD. All these measurements exhibit a similar NPV (Table 1).

Another interesting point regards the IM nails used to fix the intertrochanteric fracture. In the present study, mobilisations occurred especially in patients in whom one type of nail had been used. To our knowledge, three studies report the association of different incidence of mobilisation following internal fixation performed using the InterTAN, characterised by two cephalic screws, and the Proximal Femoral Nail Antirotation (PFNA), characterised by only one cephalic screw, nailing systems. However, they did not evaluate the statistical association between a TAD greater than 25 mm and mobilisation, and the CalTAD is not considered in any of them [42–44]. Furthermore, a recent study showed, in a fracture model, that a lower TAD is associated with a higher biomechanical stability in both nailing systems [45]. Unfortunately, the relatively small number of patients treated with Intertan nails in the present study prevents more advanced analytical statistics.

In any case, instructing surgeons in the concept of the tip–apex distance has helped to increase the number of patients with satisfactory positioning of the lag screw [46, 47], decreasing the frequency of cephalic screw mobilisation [46].

## Conclusion

The present study stresses the importance of accurate surgical technique to prevent cephalic screw and nail mobilisation. If revision surgery becomes necessary, it may be complicated by a high mortality risk, or, at the very least, a high rate of in-hospital morbidity and costs. Surgeons should strive for a TAD and CalTAD less than 25 mm, and a TADcalTAD less than 50 mm when using intramedullary devices, especially in the management of unstable intertrochanteric hip fractures to prevent mobilisation of the lag screw. It remains to be proven whether the TADcalTAD is equally important when a sliding hip screw construct is used.

## Abbreviations

AP: Anteroposterior
BMD: Bone mineral density
CalTAD: Calcar referenced TAD
CM: Cephalomedullary
DHS: Dynamic hip screw
IM: Intramedullary
IT: Intertrochanteric
NPV: Negative predictive value
PFNA: Proximal femoral nail antirotation
PPV: Positive predictive value
SHS: Sliding hip screws
ST: Subtrochanteric
TAD: Tip–apex distance
